# Brain energy metabolism as an underlying basis of slow and fast cognitive phenotypes in honeybees

**DOI:** 10.1242/jeb.247835

**Published:** 2024-09-03

**Authors:** Catherine Tait, Adam J. Chicco, Dhruba Naug

**Affiliations:** ^1^ Department of Biology, Colorado State University, Fort Collins, CO 80523, USA; ^2^ Department of Biomedical Sciences, Colorado State University, Fort Collins, CO 80523, USA

**Keywords:** Cognitive phenotypes, Slow–fast variation, Speed accuracy trade-off, Metabolic rate, Brain respiration, Honeybee

## Abstract

In the context of slow–fast behavioral variation, fast individuals are hypothesized to be those who prioritize speed over accuracy while slow individuals are those which do the opposite. Since energy metabolism is a critical component of neural and cognitive functioning, this predicts such differences in cognitive style to be reflected at the level of the brain. We tested this idea in honeybees by first classifying individuals into slow and fast cognitive phenotypes based on a learning assay and then measuring their brain respiration with high-resolution respirometry. Our results broadly show that inter-individual differences in cognition are reflected in differences in brain mass and accompanying energy use at the level of the brain and the whole animal. Larger brains had lower mass-specific energy usage and bees with larger brains had a higher metabolic rate. These differences in brain respiration and brain mass were, in turn, associated with cognitive differences, such that bees with larger brains were fast cognitive phenotypes whereas those with smaller brains were slow cognitive phenotypes. We discuss these results in the context of the role of energy in brain functioning and slow–fast decision making and speed accuracy trade-off.

## Teaser

**Highlighted Article:** Honeybees with fast cognitive phenotypes, prioritizing speed over accuracy, have larger brains with a lower mass-specific brain respiration rate, relative to slow cognitive phenotypes, which prioritize accuracy over speed.

## INTRODUCTION

Slow–fast phenotypic differences in terms of behavior, life history and physiology, integrated into a suite of traits described as the ‘pace of life’, have lately attracted considerable attention (Ricklefs and Wikelski, 2002; Réale et al., 2010). Metabolic rate, the rate at which organisms acquire, transform and expend energy, and therefore often described as the fundamental biological rate (Brown et al., 2004), has been cited as the potential pacemaker that drives this slow–fast phenotypic variation (Glazier, 2015; Biro and Stamps, 2010). Although there is substantial empirical support for an association between metabolic rate and slow–fast phenotypic differences, both between and within species (Wiersma et al., 2007; Pettersen et al., 2016; Wong et al., 2021), the mechanistic relationship between metabolic rate and behavioral traits is complex and far from clear (Careau et al., 2008; Salzman et al., 2018; Chung et al., 2018). In this context, it has also been proposed that such slow–fast differences are fundamentally tied to cognitive differences related to a speed–accuracy trade-off in decision-making, in which fast phenotypes spend less time gathering information, making rapid but less accurate decisions, compared with slow phenotypes (Carere and Locurto, 2011; Sih and Del Guidice, 2012; Dougherty and Guillette, 2018).

Both behavioral ecology and neuroscience research have independently emphasized the role of energy metabolism in different cognitive processes and in the modulation of complex behavioral phenotypes (Niven and Laughlin, 2008; Mathot and Dingemanse, 2015), but few studies have integrated this perspective to understand the underpinnings of behavioral variation (Rittschof et al., 2015; Coto and Traniello, 2021). Neural tissue is widely known to be energetically expensive to produce, operate and maintain (Laughlin et al., 1998; Laughlin, 2001; Ames, 2000; Lennie, 2003). This is substantiated by a large body of work demonstrating that cognitive processes such as learning and memory formation are associated with significant energetic costs (Déglise et al., 2003; Mery and Kawecki, 2005; Jaumann et al., 2013; Plaçais and Preat, 2013). There is also substantial inter-individual variation in energy production and usage in the brain that has, in turn, been linked to behavioral variation, notably in social species (Hollis et al., 2015; Rittschof and Schirmeier, 2018).

In social insects, behavioral variation – the underlying basis for division of labor – has been shown to be correlated to differences in brain organization (Molina et al., 2009; Muscedere and Traniello, 2014; Kamhi et al., 2016). More specifically, in terms of cognitive differences, slow–fast differences in behavior were shown to match the predicted differences in metabolic rate and cognitive traits in honeybees (Mugel and Naug, 2020; Tait and Naug, 2020). This provides a background to test whether slow and fast cognitive phenotypes in honeybees exhibit any differences in the energetic capacity of their brains. In this study, we therefore first classified bees into slow and fast cognitive types using a discrimination and reversal learning assay based on the principle that fast phenotypes are defined by fast discrimination learning and slow reversal learning while the opposite is true for slow phenotypes (Sih and Del Giudice, 2012). We then measured the whole-animal metabolic rate of these bees using flow-through respirometry, followed by measuring the maximum oxidative phosphorylation (OXPHOS)-linked respiration rate of their brain using high-resolution respirometry. The broad goal of the study was to test the hypothesis that slow–fast cognitive differences and the related speed–accuracy trade-off are associated with differences in energy metabolism at the level of the brain, which in turn is reflected in differences in brain size and whole-animal metabolic rate.

## MATERIALS AND METHODS

The bees used in the experiment came from six source colonies of the honeybee *Apis mellifera*. Brood frames with pupae were collected one day prior to adult emergence and kept in an incubator set at 32°C. Upon emergence, individual adult bees were marked with paint on their thorax and introduced into an experimental hive that consisted of two brood frames, a full honey frame, a laying queen and workers. Marked bees of foraging age were collected at the hive entrance, immobilized on ice, and harnessed within a 4.5 cm long plastic drinking straw with a small wire around the thorax. Each bee was fed to satiation with 30% sucrose solution and then kept starved for 24 h inside an incubator set at 27°C to increase motivation for appetitive learning. Before the start of the learning assay, all bees were tested for their responsiveness to sucrose by touching their antennae with 30% sucrose solution and any bee that did not extend its proboscis to this stimulus was excluded.

### Discrimination and reversal learning

The discrimination and reversal learning ability of an individual bee were determined by using the proboscis extension reflex (PER) assay. This consisted of presenting a bee with an odor A (CS+) followed by a sucrose reward (US) and a second odor B (CS-) followed by a saline punishment in a predetermined pseudorandom sequence (ABBABABBABABAABAABAB) in a series of 12 trials with a 5 min inter-trial interval. Discrimination learning ability of a bee was measured first by pairing one odor with a sucrose reward (A+) and another odor with saline solution (B-) and, after a gap of 60 min, reversal learning ability of the same individual was assessed by reversing the odor pairings (A- and B+). The conditioned response of a bee, the extension of the proboscis to the CS alone prior to the presentation of US, to the sugar reward and its non-response to the saline solution were considered as correct choices and the opposite responses were considered as incorrect choices. Bees that completed both the learning assays were kept in an incubator at 27°C for 30 min before measuring their metabolic rate.

### Whole-animal metabolic rate

Active whole-animal metabolic rate (MR) of a bee was measured using flow through respirometry. Ambient air, scrubbed of H_2_O and CO_2_, was passed at a constant rate of 750 ml min^−1^ through a 250 ml sealed glass chamber containing a single bee and CO_2_ in the excurrent airstream was measured for 10 min with a FoxBox gas analyzer (Sable Systems), lightly agitating the chamber to stimulate flight as necessary. Bees that did show flight activity were discarded. Each bee was weighed immediately afterward and MR was calculated as the weight corrected mean CO_2_ production (in ml h^−1^ g^−1^) for the 180 s with the lowest variance in CO_2_ production. This was transformed into a weight-corrected power output (in mW g^−1^) by multiplying it with 21.4 J ml^−1^ CO_2_ and dividing by 3600 J h^−1^ W^−1^ (Mugel and Naug, 2020).

### Brain mitochondrial respiration

Following the measurement of whole-animal metabolic rate, bees were immediately anaesthetized on ice and their heads were removed. Fresh brains were carefully removed under a dissecting microscope in chilled MiR05 respiration buffer (containing 0.5 mmol l^−1^ EGTA, 3 mmol l^−1^ MgCl_2_, 60 mmol l^−1^ K-lactobionate, 20 mmol l^−1^ taurine, 10 mmol l^−1^ KH_2_PO_4_, 20 mmol l^−1^ HEPES, 110 mmol l^−1^ sucrose and 1 mmol l^−1^ g l^−1^ fatty-acid free BSA). Brains were then transferred to pre-weighed centrifuge tubes containing 2 ml chilled MiR05 buffer and weighed on a microbalance with a resolution of 0.01 mg (Mettler Toledo). Four to six brains were measured in any one round of the assay, which ensured that their oxygen consumption could be quantified simultaneously within 3–4 h of dissection, which is well within the timeframe in which fresh brain preparations remain viable (Williamson and Hiesinger, 2010; Neville et al., 2018).

Oxygen consumption rate in the brain was quantified *ex vivo* using an Oxygraph-2k high-resolution respirometer (Oroboros Instruments GmbH, Innsbruck, Austria). Before adding brains, chambers were rinsed with 70% ethanol (3×) and milliQ H_2_O (6×), then filled with MiR05 respiration buffer and air-calibrated to a starting oxygen content of 160 μM in a circulated chamber (with a 750 r.p.m. stir bar) maintained at 37°C. Following measurement of basal oxygen consumption rate of intact brains, digitonin (10 µg ml^−1^) was added to permeabilize brain cells while leaving mitochondrial membranes intact for assessments of substrate-specific respiratory capacities. Low flux LEAK respiration state was measured first by adding a combination of substrates (0.5 mmol l^−1^ malate, 5 mmol l^−1^ pyruvate and 10 mmol l^−1^ glutamate) in the absence of ADP, which reflect NADH-linked electron flow facilitated by non-specific proton leak across the inner mitochondrial membrane. The OXPHOS-linked respiration rate was measured next by the addition of 2.5 mmol l^−1^ ADP to the same chamber, which enables a higher rate of NADH-linked electron flow by dissipating the inner membrane proton gradient through the ATP synthase. Finally, a maximal OXPHOS-linked respiration rate was measured by the addition of 20 mmol l^−1^ succinate to provide additional electron supply through succinate dehydrogenase (CII), thereby fully reconstituting the supply of reducing equivalents from the tricarboxylic acid cycle to the electron transfer system. During these measurements, mass-corrected oxygen consumption rates were recorded continuously by monitoring changes in the negative time derivative of the chamber oxygen concentration signal following standardized instrumental and chemical background calibrations and recorded as the average of at least 2 min of stable (linear) oxygen consumption readings. Brain energetic capacity was defined in terms of mass-specific brain respiration, calculated from the maximum OXPHOS-linked respiration, a direct indicator of mitochondrial bioenergetics (Sauerbeck et al., 2011; Rittschof et al., 2018).

### Statistical analysis

Discrimination and reversal learning scores of a bee were calculated as:
(1)

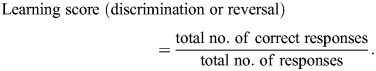


Slow and fast cognitive phenotypes were defined based on a learning index that was calculated for each bee by subtracting its discrimination learning score from its reversal learning score (reversal score–discrimination score). This resulted in values ranging from −1 to +1, where individuals with negative indices defined fast cognitive phenotypes (low reversal and high discrimination scores) and individuals with positive indices defined slow cognitive phenotypes (high reversal and low discrimination scores). The association among the different traits was assessed using the linear mixed model package lme4 in R (https://github.com/lme4/lme4/) with age and colony of origin used as random effects.

All data used in this research are available in Dataset 1.

## RESULTS

**Fig. 1.**
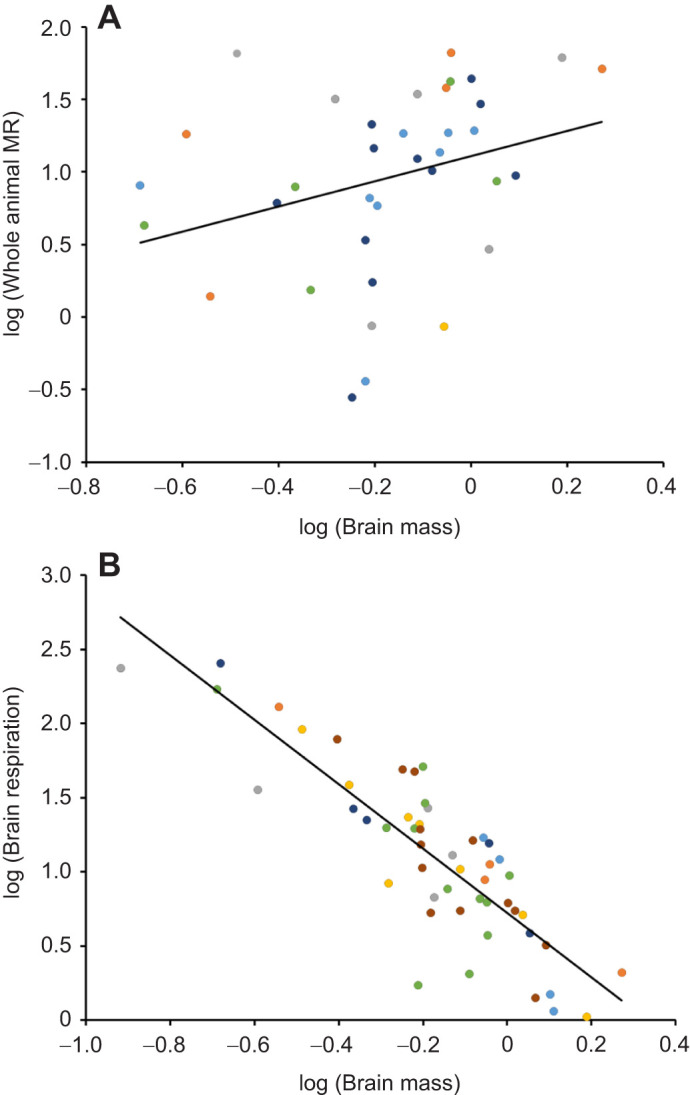
**Whole-animal metabolic rate and mitochondrial respiration in the brain of honeybees as a function of brain mass.** Log–log plots of (A) whole-animal metabolic rate (MR) (mW) and (B) brain mitochondrial respiration (pmol O_2_ mg^−1^ s^−1^) as a function of brain mass (mg). Datapoints of different colors represent individual bees from different colonies and the lines represent the direction of significant relationships (A, *N*=37; B, *N*=51).

Brain mass of bees was positively correlated with mass-specific whole-animal metabolic rate (χ^2^=5.52, *N*=37, *P*=0.01, Fig. 1A) and negatively correlated with mass-specific brain mitochondrial respiration (χ^2^=44.58, *N*=51, *P*<0.001, Fig. 1B). There was, however, no significant relationship between brain mitochondrial respiration and whole-animal metabolic rate (χ^2^=1.34, *N*=51, *P*=0.24, Table S1).

**Fig. 2.**
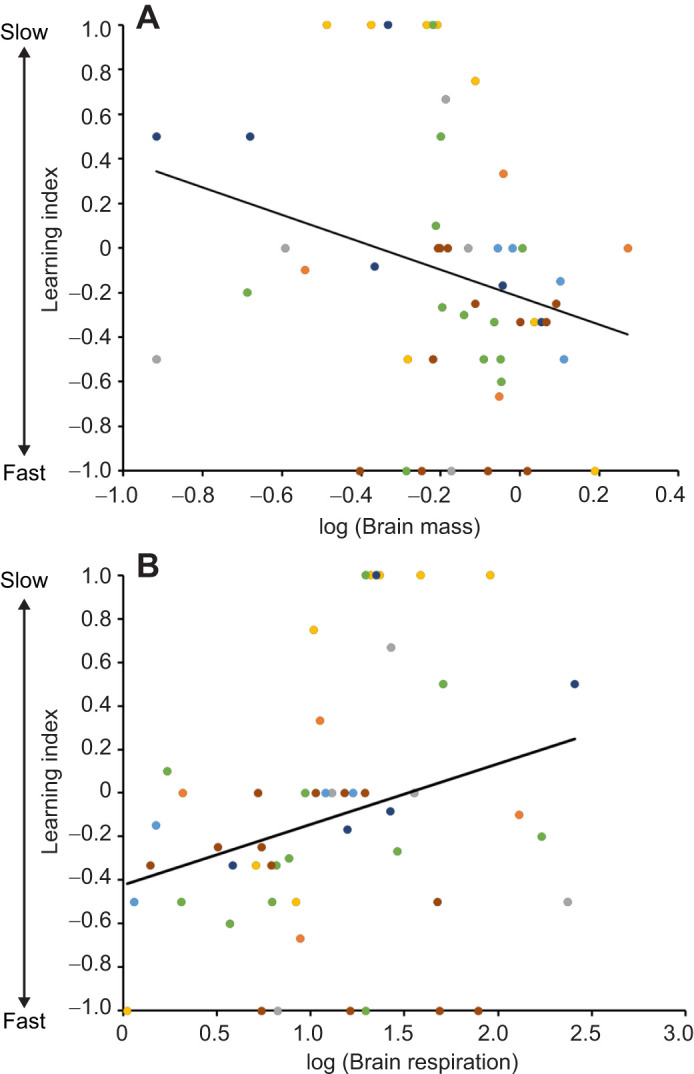
**Honeybee cognitive phenotype as a function of brain mass and brain mitochondrial respiration.** Cognitive function versus log (A) brain mass (mg) and (B) brain mitochondrial respiration (pmol O_2_ mg^−1^ s^−1^). A negative learning index defines fast cognitive phenotypes and a positive learning index defines slow cognitive phenotypes. Data points of different colors represent bees (*N*=51) from different colonies and the lines represent the direction of significant relationships.

The learning index of a bee was negatively correlated to brain mass (χ^2^=4.94, *N*=51, *P*=0.02, Fig. 2A) but positively correlated to brain mitochondrial respiration (χ^2^=4.43, *P*=0.01, *N*=51, Fig. 2B), which means that bees with fast cognitive phenotypes had larger brains with a lower respiratory rate compared with slow bees. Learning index, however, was not significantly correlated to whole-animal metabolic rate (χ^2^=0.14, *N*=51, *P*=0.7). There was no effect of age or colony of origin on any of the relationships (Table S1).

## DISCUSSION

This is the first time, to the best of our knowledge, slow–fast differences in cognition have been shown to be associated with differences in brain respiration rate, thereby establishing a possible common energetic link that ties the cognitive axis with other parts of the pace-of-life axis. Fast bees were those with larger brains, which were also associated with an overall higher whole-animal metabolic rate compared with slow bees. The observed positive relationship between brain mass and whole-animal metabolic rate is consistent with what has been observed across a wide variety of taxa (Isler and van Schaik, 2006) and can be attributed to the high energetic expense of brain tissue (Mink et al., 1981; Ames, 2000). While the larger brains seem to impose an overall higher metabolic expenditure in the fast bees, it is important to note that the larger brains were also associated with lower mass-specific energy consumption. It has been shown that there is a hypometric relationship in terms of how energy consumption of the brain scales with brain mass (Karbowski, 2007), which along with other mechanisms such as reduced size of other metabolically expensive tissues (Aiello and Wheeler, 1995; Goncerzewicz, et al., 2022) can lower the relative cost of larger brains.

The allometric relationship of larger brains having relatively lower rates of energy usage is generally attributed to decreases in the density of neurons or their firing rate (Karbowski, 2007, 2009). This suggests that differences in brain size could be associated with possible trade-offs in cognitive performance. A speed–accuracy trade-off is an intrinsic part of cognitive performance and fast cognitive phenotypes are defined as those which prioritize speed over accuracy, while slow cognitive phenotypes are those which show the opposite pattern (Sih and Del Giudice, 2012). There is ample evidence of such alternative cognitive strategies in honeybees (Chittka et al., 2003; Burns and Dyer, 2008; Tait and Naug, 2020; 2022) and the results from this study suggest that fast bees are those with larger brains with a high overall but low mass-specific energy consumption. The only other study which we found to have tested for a similar relationship observed a somewhat opposite pattern in guppies such that there was a positive correlation between telencephalon size and the time to make decision, although its relationship with accuracy was less clear (Burns and Rodd, 2008). This would mean that fishes with larger brains are likely to have slow cognitive phenotypes compared with those with smaller brains. However, this study did not measure energy usage in the brain, making it difficult to directly compare their results with what was found here. It also suggests that brain size alone may not be enough to explain how it influences cognitive functioning. Although brain size has often been used as a measure of cognitive capacity (Deaner et al., 2007; Benson-Amram et al., 2016; Collado et al., 2021), it has also repeatedly been shown that the relationship between the two is more complex (Schoenemann et al., 2000; Chittka and Niven, 2009; Logan et al., 2018; Triki et al., 2021; Hooper et al., 2022) and energetic considerations are an important part of it (Herculano-Houzel, 2011; Heldstab et al., 2022).

Energy used in the brain is largely required for the propagation of action potentials and for restoring postsynaptic ion fluxes, and the energetic cost of information processing at the neuronal level is associated with noise and the speed of response (Laughlin et al., 1998; Laughlin, 2001; Niven et al., 2007). During signal transmission, speed is related to the bandwidth over which a signal is transmitted while accuracy is related to the signal-to-noise ratio. Noise can be reduced by averaging the outputs from multiple neurons, although it comes with an increased energetic cost. Therefore, the number of neurons that can be activated will trade off with the average discharge rate of each neuron. Signal-to-noise ratios can also be improved by a higher level of inhibition at the neuronal level that results in slower neuronal integration of the downstream process. The fixed and signaling cost of a neuron both increase supra-linearly with its ability to transmit information and therefore efficiency declines with increasing capacity. It has been shown that while energy usage is positively correlated to the transmission rate of neuronal signals, cells that fire at a higher rate also carry less information (Ames, 2000; Koch et al., 2006). The cost of each spike puts a constraint on how many neurons can be concurrently active, thus implying an upper limit to aggregate neural activity and task accuracy (Levy and Baxter, 1996).

Models of speed–accuracy trade-off in decision-making are largely accumulator models that are based on two parameters: information accumulation and a threshold that defines the amount of information at which a decision is made (Gold and Shadlen, 2007; Bogacz et al., 2010; Heitz and Schall, 2012; Standage et al., 2014). This broadly implies that adjustments in either the rate of information accumulation, or the value of the decision threshold, or both, are at the basis of speed accuracy trade-off. The rate of information accumulation can be directly tied to the firing rate of individual neurons and/or the total number of neurons involved in the process, which in turn will be correlated to the overall energy expenditure (Penconek, 2022). A higher firing rate would allow a given value of decision threshold to be reached sooner, which might suggest that a higher level of energy metabolism in the brain is associated with fast cognitive phenotypes. However, an alternative possibility is that brains with lower rates of energy metabolism are associated with lower decision thresholds, which means that they can reach decisions sooner, consistent with what we report here. There is some evidence that information processing speed is lower during energetic compromise, and hungry neurons cannot maintain the firing speed necessary for optimal computations (Lord et al., 2013; Killeen et al., 2016). While faster decisions can be made by either increasing the rate of information accumulation or lowering the decision threshold, a higher accuracy is more constrained by a high value of the decision threshold and therefore by the quality and efficiency of information transfer in which energetic considerations play a strong role (Schreiber et al., 2002; Lennie, 2003). Our results suggest a higher energetic capacity in the brain might be needed to achieve these higher thresholds that translate to the higher accuracy defining the slow cognitive phenotypes.

Our measurement of brain respiration specifically reflects oxidative phosphorylation and mitochondrial activity, which are particularly important in meeting neuronal energetic demands (Kasischke et al., 2004; Ly and Verstreken, 2006; Hall et al., 2012; Picard and McEwen, 2014). It has been proposed that mitochondrial respiratory capacity could play a key role in the phenotypic variation related to pace of life (Jimenez et al., 2014), but the empirical evidence for this is somewhat mixed, with slow–fast differences being correlated to mitochondrial activity in the liver, but not in the brain or the heart (Chung et al., 2018). This suggests that the connection between mitochondrial function and whole-organism metabolic rate, and thus pace of life, is likely to be complicated. The plasticity of mitochondrial function both in the short-term and across life stages adds a further level of complexity to this issue (Chan, 2006; Jendrach et al., 2008). In addition, one may need to account for differences in the organizational structure of the neurocircuitry, which has a strong role in determining the efficiency of information transfer and energy usage in the brain (Chittka and Niven, 2009; Sengupta et al., 2013; Karbowski, 2019; Farnworth and Montgomery, 2024). Energy metabolism is a critical component of cognitive function and many cognitive impairments are associated with metabolic dysfunction (Sullivan et al., 2005; Lin and Beal, 2006; Frisardi et al., 2010), underscoring the importance of understanding how energetics and metabolism are associated with interindividual differences in cognition.

Slow and fast cognitive phenotypes represent differences in information acquisition strategy (Sih and Del Giudice, 2012; Tait and Naug, 2020, 2022). It has been shown that such differences could be tied to physiological differences (Moreira et al., 2004; Øverli et al., 2007) although our understanding of the underlying mechanisms that drive differences between cognitive types is still limited. The foundational idea of cognitive ability being tied to brain size and accompanying energetic demands (Dunbar and Shultz, 2007; Pérez-Barbería et al., 2007) has not been extended to understand the basis of slow–fast cognitive variation at the individual level. More recently, it has been shown how behavioral variation can be traced to energetic processes at the brain level (Chandrasekaran et al., 2015; Hollis et al., 2015; Kamhi et al., 2016; Rittschof et al., 2018), which provides support for the idea that differences in metabolic rate and energetic capacity may be the common underlying basis of variation across multiple phenotypic levels.

## Supplementary Material

10.1242/jexbio.247835_sup1 Supplementary information

Dataset 1. Brain energy metabolism

## References

[JEB247835C1] Aiello, L. C. and Wheeler, P. (1995). The expensive-tissue hypothesis: the brain and the digestive system in human and primate evolution. *Curr. Anthropol.* 36, 199-221. 10.1086/204350

[JEB247835C2] Ames, A. (2000). CNS energy metabolism as related to function. *Brain Res. Rev.* 34, 42-68. 10.1016/S0165-0173(00)00038-2 11086186

[JEB247835C3] Benson-Amram, S., Dantzer, B., Stricker, G., Swanson, E. M. and Holekamp, K. E. (2016). Brain size predicts problem-solving ability in mammalian carnivores. *Proc. Natl Acad. Sci. USA* 113, 2532-2537. 10.1073/pnas.1505913113 26811470 PMC4780594

[JEB247835C4] Biro, P. A. and Stamps, J. A. (2010). Do consistent individual differences in metabolic rate promote consistent individual differences in behavior? *Trends Ecol. Evol.* 25, 653-659. 10.1016/j.tree.2010.08.003 20832898

[JEB247835C5] Bogacz, R., Wagenmakers, E.-J., Forstmann, B. U. and Nieuwenhuis, S. (2010). The neural basis of the speed–accuracy tradeoff. *Trends Neurosci.* 33, 10-16. 10.1016/j.tins.2009.09.002 19819033

[JEB247835C6] Brown, J. H., Gillooly, J. F., Allen, A. P., Savage, V. M. and West, G. B. (2004). Toward a metabolic theory of ecology. *Ecology* 85, 1771-1789. 10.1890/03-9000

[JEB247835C7] Burns, J. G. and Dyer, A. G. (2008). Diversity of speed-accuracy strategies benefits social insects. *Curr. Biol.* 18, R953-R954. 10.1016/j.cub.2008.08.028 18957249

[JEB247835C8] Burns, J. G. and Rodd, F. H. (2008). Hastiness, brain size and predation regime affect the performance of wild guppies in a spatial memory task. *Anim. Behav.* 76, 911-922. 10.1016/j.anbehav.2008.02.017

[JEB247835C9] Careau, V., Thomas, D., Humphries, M. M. and Réale, D. (2008). Energy metabolism and animal personality. *Oikos* 117, 641-653.

[JEB247835C10] Carere, C. and Locurto, C. (2011). Interaction between animal personality and animal cognition. *Curr. Zool.* 57, 491-498. 10.1093/czoolo/57.4.491

[JEB247835C11] Chan, D. C. (2006). Mitochondria: dynamic organelles in disease, aging, and development. *Cell* 125, 1241-1252. 10.1016/j.cell.2006.06.010 16814712

[JEB247835C12] Chandrasekaran, S., Rittschof, C. C., Djukovic, D., Gu, H., Raftery, D., Price, N. D. and Robinson, G. E. (2015). Aggression is associated with aerobic glycolysis in the honey bee brain. *Genes Brain Behav.* 14, 158-166. 10.1111/gbb.12201 25640316 PMC4449359

[JEB247835C13] Chittka, L. and Niven, J. (2009). Are bigger brains better? *Curr. Biol.* 19, R995-R1008. 10.1016/j.cub.2009.08.023 19922859

[JEB247835C14] Chittka, L., Dyer, A. G., Bock, F. and Dornhaus, A. (2003). Bees trade off foraging speed for accuracy. *Nature* 424, 388-388. 10.1038/424388a 12879057

[JEB247835C15] Chung, D. J., Healy, T. M., McKenzie, J. L., Chicco, A. J., Sparagna, G. C. and Schulte, P. M. (2018). Mitochondria, temperature, and the pace of life. *Integr. Comp. Biol.* 58, 578-590. 10.1093/icb/icy013 29718252

[JEB247835C16] Collado, M. Á., Montaner, C. M., Molina, F. P., Sol, D. and Bartomeus, I. (2021). Brain size predicts learning abilities in bees. *R. Soc. Open Sci.* 8, 201940. 10.1098/rsos.201940 34017597 PMC8131939

[JEB247835C17] Coto, Z. N. and Traniello, J. F. A. (2021). Brain size, metabolism, and social evolution. *Front. Physiol.* 12, 612865. 10.3389/fphys.2021.612865 33708134 PMC7940180

[JEB247835C18] Deaner, R. O., Isler, K., Burkart, J. and van Schaik, C. (2007). Overall brain size, and not encephalization quotient, best predicts cognitive ability across non-human primates. *Brain Behav. Evol.* 70, 115-124. 10.1159/000102973 17510549

[JEB247835C19] Déglise, P., Dacher, M., Dion, E., Gauthier, M. and Armengaud, C. (2003). Regional brain variations of cytochrome oxidase staining during olfactory learning in the honeybee (*Apis mellifera*). *Behav. Neurosci.* 117, 540-547. 10.1037/0735-7044.117.3.540 12802882

[JEB247835C20] Dougherty, L. R. and Guillette, L. M. (2018). Linking personality and cognition: a meta-analysis. *Philos. Trans. R. Soc. B: Biol. Sci.* 373, 20170282. 10.1098/rstb.2017.0282 PMC6107561 30104427

[JEB247835C21] Dunbar, R. I. M. and Shultz, S. (2007). Evolution in the social brain. *Science* 317, 1344-1347. 10.1126/science.1145463 17823343

[JEB247835C22] Farnworth, M. S. and Montgomery, S. H. (2024). Evolution of neural circuitry and cognition. *Biol. Lett.* 20, 20230576. 10.1098/rsbl.2023.0576 38747685 PMC11285921

[JEB247835C23] Frisardi, V., Solfrizzi, V., Seripa, D., Capurso, C., Santamato, A., Sancarlo, D., Vendemiale, G., Pilotto, A. and Panza, F. (2010). Metabolic-cognitive syndrome: a cross-talk between metabolic syndrome and Alzheimer's disease. *Ageing Res. Rev.* 9, 399-417. 10.1016/j.arr.2010.04.007 20444434

[JEB247835C24] Glazier, D. S. (2015). Is metabolic rate a universal ‘pacemaker’ for biological processes? *Biol. Rev.* 90, 377-407. 10.1111/brv.12115 24863680

[JEB247835C25] Gold, J. I. and Shadlen, M. N. (2007). The neural basis of decision making. *Annu. Rev. Neurosci.* 30, 535-574. 10.1146/annurev.neuro.29.051605.113038 17600525

[JEB247835C26] Goncerzewicz, A., Górkiewicz, T., Dzik, J. M., Jędrzejewska-Szmek, J., Knapska, E. and Konarzewski, M. (2022). Brain size, gut size and cognitive abilities: the energy trade-offs tested in artificial selection experiment. *Proc. R. Soc. B* 289, 20212747. 10.1098/rspb.2021.2747 PMC9006030 35414242

[JEB247835C27] Hall, C. N., Klein-Flügge, M. C., Howarth, C. and Attwell, D. (2012). Oxidative phosphorylation, not glycolysis, powers presynaptic and postsynaptic mechanisms underlying brain information processing. *J. Neurosci.* 32, 8940-8951. 10.1523/JNEUROSCI.0026-12.2012 22745494 PMC3390246

[JEB247835C28] Heitz, R. P. and Schall, J. D. (2012). Neural mechanisms of speed-accuracy tradeoff. *Neuron* 76, 616-628. 10.1016/j.neuron.2012.08.030 23141072 PMC3576837

[JEB247835C29] Heldstab, S. A., Isler, K., Graber, S. M., Schuppli, C. and van Schaik, C. P. (2022). The economics of brain size evolution in vertebrates. *Curr. Biol.* 32, R697-R708. 10.1016/j.cub.2022.04.096 35728555

[JEB247835C30] Herculano-Houzel, S. (2011). Scaling of brain metabolism with a fixed energy budget per neuron: implications for neuronal activity, plasticity and evolution. *PLoS One* 6, e17514. 10.1371/journal.pone.0017514 21390261 PMC3046985

[JEB247835C31] Hollis, F., van der Kooij, M. A., Zanoletti, O., Lozano, L., Cantó, C. and Sandi, C. (2015). Mitochondrial function in the brain links anxiety with social subordination. *Proc. Natl Acad. Sci. USA* 112, 15486-15491. 10.1073/pnas.1512653112 26621716 PMC4687564

[JEB247835C32] Hooper, R., Brett, B. and Thornton, A. (2022). Problems with using comparative analyses of avian brain size to test hypotheses of cognitive evolution. *PLoS One* 17, e0270771. 10.1371/journal.pone.0270771 35867640 PMC9307164

[JEB247835C33] Isler, K. and van Schaik, C. P. (2006). Metabolic costs of brain size evolution. *Biol. Lett.* 2, 557-560. 10.1098/rsbl.2006.0538 17148287 PMC1834002

[JEB247835C34] Jaumann, S., Scudelari, R. and Naug, D. (2013). Energetic cost of learning and memory can cause cognitive impairment in honeybees. *Biol. Lett.* 9, 20130149. 10.1098/rsbl.2013.0149 23784929 PMC3730623

[JEB247835C35] Jendrach, M., Mai, S., Pohl, S., Vöth, M. and Bereiter-Hahn, J. (2008). Short- and long-term alterations of mitochondrial morphology, dynamics and mtDNA after transient oxidative stress. *Mitochondrion* 8, 293-304. 10.1016/j.mito.2008.06.001 18602028

[JEB247835C36] Jimenez, A. G., Van Brocklyn, J., Wortman, M. and Williams, J. B. (2014). Cellular metabolic rate is influenced by life-history traits in tropical and temperate birds. *PLoS One* 9, e87349. 10.1371/journal.pone.0087349 24498080 PMC3907555

[JEB247835C37] Kamhi, J. F., Gronenberg, W., Robson, S. K. A. and Traniello, J. F. A. (2016). Social complexity influences brain investment and neural operation costs in ants. *Proc. R. Soc. B* 283, 20161949. 10.1098/rspb.2016.1949 PMC5095391 27798312

[JEB247835C38] Karbowski, J. (2007). Global and regional brain metabolic scaling and its functional consequences. *BMC Biol.* 5, 18. 10.1186/1741-7007-5-18 17488526 PMC1884139

[JEB247835C39] Karbowski, J. (2009). Thermodynamic constraints on neural dimensions, firing rates, brain temperature and size. *J. Comput. Neurosci.* 27, 415-436. 10.1007/s10827-009-0153-7 19415477

[JEB247835C40] Karbowski, J. (2019). Metabolic constraints on synaptic learning and memory. *J. Neurophysiol.* 122, 1473-1490. 10.1152/jn.00092.2019 31365284

[JEB247835C41] Kasischke, K. A., Vishwasrao, H. D., Fisher, P. J., Zipfel, W. R. and Webb, W. W. (2004). Neural activity triggers neuronal oxidative metabolism followed by astrocytic glycolysis. *Science* 305, 99-103. 10.1126/science.1096485 15232110

[JEB247835C42] Killeen, P. R., Russell, V. A. and Tannock, R. (2016). Neuroenergetics. *Curr. Dir. Psychol. Sci.* 25, 124-129. 10.1177/0963721416628530

[JEB247835C43] Koch, K., McLean, J., Segev, R., Freed, M. A., Berry, M. J., Balasubramanian, V. and Sterling, P. (2006). How much the eye tells the brain. *Curr. Biol.* 16, 1428-1434. 10.1016/j.cub.2006.05.056 16860742 PMC1564115

[JEB247835C44] Laughlin, S. B. (2001). Energy as a constraint on the coding and processing of sensory information. *Curr. Opin. Neurobiol.* 11, 475-480. 10.1016/S0959-4388(00)00237-3 11502395

[JEB247835C45] Laughlin, S. B., de Ruyter van Steveninck, R. R. and Anderson, J. C. (1998). The metabolic cost of neural information. *Nat. Neurosci.* 1, 36-41. 10.1038/236 10195106

[JEB247835C46] Lennie, P. (2003). The cost of cortical computation. *Curr. Biol.* 13, 493-497. 10.1016/S0960-9822(03)00135-0 12646132

[JEB247835C47] Levy, W. B. and Baxter, R. A. (1996). Energy efficient neural codes. *Neural Comput.* 8, 531-543. 10.1162/neco.1996.8.3.531 8868566

[JEB247835C48] Lin, M. T. and Beal, M. F. (2006). Mitochondrial dysfunction and oxidative stress in neurodegenerative diseases. *Nature* 443, 787-795. 10.1038/nature05292 17051205

[JEB247835C49] Logan, C. J., Avin, S., Boogert, N., Buskell, A., Cross, F. R., Currie, A., Jelbert, S., Lukas, D., Mares, R., Navarrete, A. F. et al. (2018). Beyond brain size: uncovering the neural correlates of behavioral and cognitive specialization. *Comp. Cogn. Behav. Rev.* 13, 55-89. 10.3819/CCBR.2018.130008

[JEB247835C50] Lord, L.-D., Expert, P., Huckins, J. F. and Turkheimer, F. E. (2013). Cerebral energy metabolism and the brain's functional network architecture: an integrative review. *J. Cereb. Blood Flow Metab.* 33, 1347-1354. 10.1038/jcbfm.2013.94 23756687 PMC3764392

[JEB247835C51] Ly, C. V. and Verstreken, P. (2006). Mitochondria at the synapse. *Neuroscientist* 12, 291-299. 10.1177/1073858406287661 16840705

[JEB247835C52] Mathot, K. J. and Dingemanse, N. J. (2015). Energetics and behavior: unrequited needs and new directions. *Trends Ecol. Evol.* 30, 199-206. 10.1016/j.tree.2015.01.010 25687159

[JEB247835C53] Mery, F. and Kawecki, T. J. (2005). A cost of long-term memory in *Drosophila*. *Science* 308, 1148. 10.1126/science.1111331 15905396

[JEB247835C54] Mink, J. W., Blumenschine, R. J. and Adams, D. B. (1981). Ratio of central nervous system to body metabolism in vertebrates: its constancy and functional basis. *Am. J. Physiol. Regul. Integr. Comp. Physiol.* 241, R203-R212. 10.1152/ajpregu.1981.241.3.R203 7282965

[JEB247835C55] Molina, Y., Harris, R. M. and O'Donnell, S. (2009). Brain organization mirrors caste differences, colony founding and nest architecture in paper wasps (Hymenoptera: Vespidae). *Proc. R. Soc. B* 276, 3345-3351. 10.1098/rspb.2009.0817 PMC2817177 19553252

[JEB247835C56] Moreira, P. S. A., Pulman, K. G. T. and Pottinger, T. G. (2004). Extinction of a conditioned response in rainbow trout selected for high or low responsiveness to stress. *Horm. Behav.* 46, 450-457. 10.1016/j.yhbeh.2004.05.003 15465531

[JEB247835C57] Mugel, S. G. and Naug, D. (2020). Metabolic rate shapes phenotypic covariance among physiological, behavioral, and life-history traits in honeybees. *Behav. Ecol. Sociobiol.* 74, 129. 10.1007/s00265-020-02901-5

[JEB247835C58] Muscedere, M. L., Gronenberg, W., Moreau, C. S. and Traniello, J. F. A. (2014). Investment in higher order central processing regions is not constrained by brain size in social insects. *Proc. R. Soc. B.* 281, 20140217. 10.1098/rspb.2014.0217 PMC4043091 24741016

[JEB247835C59] Neville, K. E., Bosse, T. L., Klekos, M., Mills, J. F., Weicksel, S. E., Waters, J. S. and Tipping, M. (2018). A novel ex vivo method for measuring whole brain metabolism in model systems. *J. Neurosci. Methods* 296, 32-43. 10.1016/j.jneumeth.2017.12.020 29287743 PMC5840572

[JEB247835C60] Niven, J. E. and Laughlin, S. B. (2008). Energy limitation as a selective pressure on the evolution of sensory systems. *J. Exp. Biol.* 211, 1792-1804. 10.1242/jeb.017574 18490395

[JEB247835C61] Niven, J. E., Anderson, J. C. and Laughlin, S. B. (2007). Fly photoreceptors demonstrate energy-information trade-offs in neural coding. *PLoS Biol.* 5, e116. 10.1371/journal.pbio.0050116 17373859 PMC1828148

[JEB247835C62] Øverli, Ø., Sørensen, C., Pulman, K. G. T., Pottinger, T. G., Korzan, W., Summers, C. H. and Nilsson, G. E. (2007). Evolutionary background for stress-coping styles: relationships between physiological, behavioral, and cognitive traits in non-mammalian vertebrates. *Neurosci. Biobehav. Rev.* 31, 396-412. 10.1016/j.neubiorev.2006.10.006 17182101

[JEB247835C63] Penconek, M. (2022). Computational analysis of speed-accuracy tradeoff. *Sci. Rep.* 12, 21995. 10.1038/s41598-022-26120-2 36539428 PMC9768160

[JEB247835C64] Pérez-Barbería, F. J., Shultz, S. and Dunbar, R. I. M. (2007). Evidence for coevolution of sociality and relative brain size in three orders of mammals. *Evolution* 61, 2811-2821. 10.1111/j.1558-5646.2007.00229.x 17908248

[JEB247835C65] Pettersen, A. K., White, C. R. and Marshall, D. J. (2016). Metabolic rate covaries with fitness and the pace of the life history in the field. *Proc. R. Soc. B* 283, 20160323. 10.1098/rspb.2016.0323 PMC4892794 27226476

[JEB247835C66] Picard, M. and McEwen, B. S. (2014). Mitochondria impact brain function and cognition. *Proc. Natl Acad. Sci. USA* 111, 7-8. 10.1073/pnas.1321881111 24367081 PMC3890847

[JEB247835C67] Plaçais, P.-Y. and Preat, T. (2013). To favor survival under food shortage, the brain disables costly memory. *Science* 339, 440-442. 10.1126/science.1226018 23349289

[JEB247835C68] Réale, D., Garant, D., Humphries, M. M., Bergeron, P., Careau, V. and Montiglio, P.-O. (2010). Personality and the emergence of the pace-of-life syndrome concept at the population level. *Philos. Trans. R. Soc. B: Biol. Sci.* 365, 4051-4063. 10.1098/rstb.2010.0208 PMC2992747 21078657

[JEB247835C69] Ricklefs, R. E. and Wikelski, M. (2002). The physiology/life-history nexus. *Trends Ecol. Evol.* 17, 462-468. 10.1016/S0169-5347(02)02578-8

[JEB247835C70] Rittschof, C. C. and Schirmeier, S. (2018). Insect models of central nervous system energy metabolism and its links to behavior. *Glia* 66, 1160-1175. 10.1002/glia.23235 28960551

[JEB247835C71] Rittschof, C. C., Grozinger, C. M. and Robinson, G. E. (2015). The energetic basis of behavior: bridging behavioral ecology and neuroscience. *Curr. Opin. Behav. Sci.* 6, 19-27. 10.1016/j.cobeha.2015.07.006

[JEB247835C72] Rittschof, C. C., Vekaria, H. J., Palmer, J. H. and Sullivan, P. G. (2018). Brain mitochondrial bioenergetics change with rapid and prolonged shifts in aggression in the honey bee, *Apis mellifera*. *J. Exp. Biol.* 221, 176917. 10.1242/jeb.176917 29496782

[JEB247835C73] Salzman, T. C., McLaughlin, A. L., Westneat, D. F. and Crowley, P. H. (2018). Energetic trade-offs and feedbacks between behavior and metabolism influence correlations between pace-of-life attributes. *Behav. Ecol. Sociobiol.* 72, 54. 10.1007/s00265-018-2460-3

[JEB247835C74] Sauerbeck, A., Pandya, J., Singh, I., Bittman, K., Readnower, R., Bing, G. and Sullivan, P. (2011). Analysis of regional brain mitochondrial bioenergetics and susceptibility to mitochondrial inhibition utilizing a microplate based system. *J. Neurosci. Methods* 198, 36-43. 10.1016/j.jneumeth.2011.03.007 21402103 PMC3535268

[JEB247835C75] Schoenemann, P. T., Budinger, T. F., Sarich, V. M. and Wang, W. S.-Y. (2000). Brain size does not predict general cognitive ability within families. *Proc. Natl. Acad. Sci. USA* 97, 4932-4937. 10.1073/pnas.97.9.4932 10781101 PMC18335

[JEB247835C76] Schreiber, S., Machens, C. K., Herz, A. V. M. and Laughlin, S. B. (2002). Energy-efficient coding with discrete stochastic events. *Neural Comput.* 14, 1323-1346. 10.1162/089976602753712963 12020449

[JEB247835C77] Sengupta, B., Stemmler, M. B. and Friston, K. J. (2013). Information and efficiency in the nervous system—a synthesis. *PLoS Comput. Biol.* 9, e1003157. 10.1371/journal.pcbi.1003157 23935475 PMC3723496

[JEB247835C78] Sih, A. and Del Giudice, M. (2012). Linking behavioural syndromes and cognition: a behavioural ecology perspective. *Philos. Trans. R. Soc. B: Biol. Sci.* 367, 2762-2772. 10.1098/rstb.2012.0216 PMC3427552 22927575

[JEB247835C79] Standage, D., Blohm, G. and Dorris, M. C. and Springer, J. E. (2014). On the neural implementation of the speed-accuracy trade-off. *Front. Neurosci.* 8, 236. 10.3389/fnins.2014.00236 25165430 PMC4131279

[JEB247835C80] Sullivan, P. G., Rabchevsky, A. G., Waldmeier, P. C. and Springer, J. E. (2005). Mitochondrial permeability transition in CNS trauma: cause or effect of neuronal cell death? *J. Neurosci. Res.* 79, 231-239. 10.1002/jnr.20292 15573402

[JEB247835C81] Tait, C. and Naug, D. (2020). Cognitive phenotypes and their functional differences in the honey bee, *Apis mellifera*. *Anim. Behav.* 165, 117-122. 10.1016/j.anbehav.2020.04.023

[JEB247835C82] Tait, C. and Naug, D. (2022). Interindividual variation in the use of social information during learning in honeybees. *Proc. R. Soc. B* 289, 20212501. 10.1098/rspb.2021.2501 PMC8790335 35078365

[JEB247835C83] Triki, Z., Aellen, M., van Schaik, C. P. and Bshary, R. (2021). Relative brain size and cognitive equivalence in fishes. *Brain Behav. Evol.* 96, 124-136. 10.1159/000520741 34753141

[JEB247835C84] Wiersma, P., Muñoz-Garcia, A., Walker, A. and Williams, J. B. (2007). Tropical birds have a slow pace of life. *Proc. Natl. Acad. Sci. USA* 104, 9340-9345. 10.1073/pnas.0702212104 17517640 PMC1890496

[JEB247835C85] Williamson, W. R. and Hiesinger, P. R. (2010). Preparation of developing and adult *Drosophila* brains and retinae for live imaging. *J. Vis. Exp.* 37, e1936. 10.3791/1936 PMC2926941 20231817

[JEB247835C86] Wong, S., Bigman, J. S. and Dulvy, N. K. (2021). The metabolic pace of life histories across fishes. *Proc. R. Soc. B* 288, 20210910. 10.1098/rspb.2021.0910 PMC8207558 34132114

