# Supplementary material for: Brain energy metabolism as an underlying basis of slow and fast cognitive phenotypes in honeybees
Source: J Exp Biol. 2024 Sep 3;227(17):jeb247835. doi: 10.1242/jeb.247835 (PMC11418170; doi:10.1242/jeb.247835)
Supplement: Supplementary information [file jexbio-227-247835-s1.pdf]

Table S1. Results of linear mixed models with fixed and random effects. Significant effects are in bold letters.

	Fixed Effects				Random Effects		
Response	Predictor	d.f	$\chi2$	*P*	Variable	Likelihood ratio	*P*
Maximum OXPHOS-linked respiration	Whole-Animal Metabolic Rate	1	1.34	0.24	Age	0.007	0.92
					Colony	< 0.001	0.99
**Brain Mass (mg)**	**Whole-Animal Metabolic Rate**	**1**	**5.52**	**0.01**	Age	< 0.001	0.99
					Colony	< 0.001	0.99
Learning Index	Whole-Animal Metabolic Rate	1	0.14	0.70	Age	0.69	0.40
					Colony	1.19	0.27
**Maximum OXPHOS-linked respiration**	**Brain Mass (mg)**	**1**	**44.58**	**< 0.001**	Age	0.04	0.84
					Colony	< 0.001	0.99
**Learning Index**	**Brain Mass (mg)**	**1**	**4.94**	**0.02**	Age	0.58	0.44
					Colony	1.82	0.17
**Learning Index**	**Maximum OXPHOS respiration**	**1**	**4.43**	**0.01**	Age	0.09	0.33
					Colony	1.58	0.20

Dataset 1. Brain energy metabolism

Available for download at
https://journals.biologists.com/jeb/article-lookup/doi/10.1242/jeb.247835#supplementary-data

Journal of Experimental Biology • Supplementary information
